# 安罗替尼治疗*KRAS*突变型晚期肺腺癌1例

**DOI:** 10.3779/j.issn.1009-3419.2018.05.13

**Published:** 2018-05-20

**Authors:** 雨栋 苏, 昭婷 孟, 晓燕 徐, 心悦 王, 冉 左, 云霞 侯, 凯 李, 鹏 陈

**Affiliations:** 300060 天津，天津医科大学肿瘤医院肺部肿瘤内科，国家肿瘤临床医学研究中心，天津市肿瘤防治重点实验室，天津市恶性肿瘤临床医学研究中心 Department of Thoracic Oncology, Tianjin Medical University Cancer Institute and Hospital, National Clinical Research Center for Cancer, Tianjin Key Laboratory of Cancer Prevention and Therapy, Tianjin Clinical Research Center for Cancer, Tianjin 300060, China

**Keywords:** 安罗替尼(Anlotinib), 肺肿瘤, 晚期肺癌, Anlotinib, Lung neoplasms, Advanced lung cancer

## Abstract

近年来，晚期非小细胞肺癌（non-small cell lung cancer, NSCLC）患者逐渐增多，治疗方法也明显增多，然而，目前对于靶向治疗表皮生长因子受体（epidermal growth factor receptor, EGFR）/间变性淋巴瘤激酶（anaplastic lymphoma kinase, ALK）耐药或化疗失败的三线及以上患者，国内外并没有标准的治疗方案，临床治疗效果也不尽如人意，安罗替尼是一种新型小分子多靶点酪氨酸激酶抑制剂，可强效抑制血管内皮细胞生长因子受体（vascular endothelial growth factor receptor, VEGFR）、血小板衍生生长因子受体（platelet-derived growth factor receptor, PDGFR）、纤维母细胞生长因子受体（fibroblast growth factor receptor, FGFR）和c-Kit等多个靶点，ALTER0303是一项关于安罗替尼作为晚期NSCLC三线治疗方案的临床研究，结果显示安罗替尼能显著延长晚期NSCLC患者的总生存期（overall survival, OS）和无进展生存期（progression-free survival, PFS），本文报道安罗替尼治疗KRAS突变型晚期肺腺癌1例。

## Abstract (en)

In recent years, the number of advanced non-small cell lung cancer (NSCLC) patients has gradually increased, and the treatment methods have also been significantly increased. However, there are no standard treatment plans at home and abroad for third-line and above patients who are refractory to targeted therapy epidermal growth factor receptor (EGFR)/anaplastic lymphoma kinase (ALK) or chemotherapy. The clinical treatment effect is also not satisfactory. Anlotinib is a novel TKI targeting the vascular endothelial growth factor receptor (VEGFR), fibroblast growth factor receptor (FGFR), platelet-derived growth factor receptor (PDGFR) and c-Kit. ALTER0303 trail, phase Ⅲ study has demonstrated that Anlotinib significantly prolonged overall survival (OS) and progression-free survival (PFS) in advanced NSCLC patients as 3^rd^ line treatment.Here we report a case of advanced lung adenocarcinoma harboring KRAS mutation treated with Anlotinib.

## 1 临床资料

患者男性，70岁，无吸烟史。因“咳嗽、咳痰、痰中带血3天”于2012年12月就诊于天津医科大学肿瘤医院。胸部计算机断层扫描（computed tomography, CT）检查提示：右下肺门占位，考虑中心性肺癌，右下肺小结节，考虑肺转移瘤，纵膈内肿大淋巴结。余检查未见明显异常。气管镜咬检病理示：腺癌（图 1）；基因检测：表皮生长因子受体（epidermal growth factor receptor, EGFR）野生型，间变性淋巴瘤激酶融合基因（anaplastic lymphoma kinase, ALK）重排阴性（Ventana D5F3 IHC），鼠类肉瘤病毒癌基因（KRAS）第2外显子突变。诊断为：右下肺癌Ⅲb期（cT4N2M0）。患者于2012年12月开始行一线紫杉醇+卡铂化疗4周期。2013年4月疗效评价病变稳定（stable disease, SD），并行右肺肿物及纵膈肿大淋巴结放疗，DT：60 Gy。2013年6月胸部CT检查提示左肺多发小结节，考虑转移。疗效评价病变进展（progressive disease, PD）。更改分期为Ⅳ期（cT4N2M1a）。2013年7月行二线多西他赛单药化疗2周期。2013年9月复查胸CT示右肺结节较前增多，左肺结节较前增大，疗效评价PD。至2015年1月，更换三线培美曲塞（PEM）+铂类化疗14周期及单药PEM维持化疗2周期。最佳疗效评价部分缓解（partial response, PR），2015年2月复查胸CT示右肺结节较前增大，疗效评价PD。2015年3月行肺部转移灶姑息放疗，DT：60 Gy。2015年11月复查胸CT示两肺多发结节较前增大，疗效评价PD（图 2A-图 2C），并入组安罗替尼临床研究，2周期后达最佳疗效PR，靶病灶体积减小33.3%并伴有肺空洞形成（图 2E）。患者耐受情况好，曾出现一过性Ⅰ度转氨酶、血压、血糖升高，对症治疗后3周缓解。2017年8月疗效评价PD，靶病灶体积增大32%（图 2H），无进展生存期（progression-free survival, PFS）达21个月余，现患者继续口服安罗替尼治疗，目前仍在随访中。

**1:**
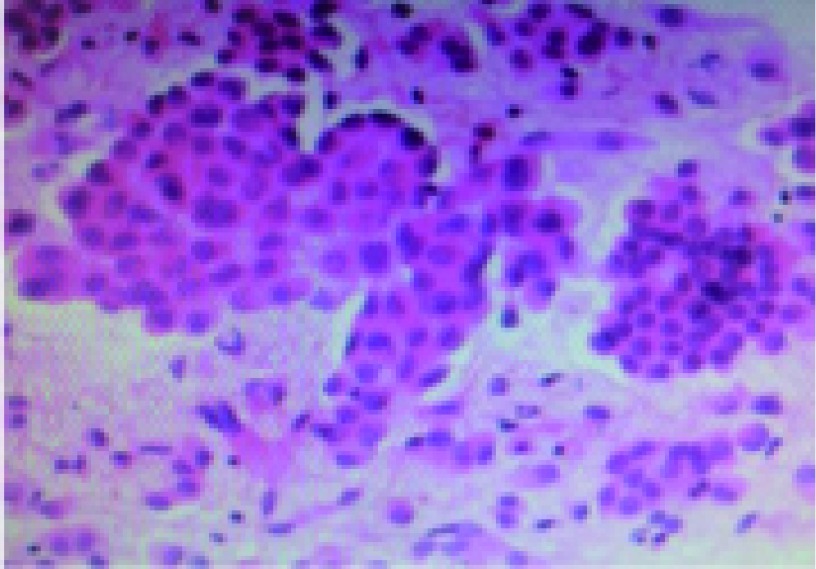
肿瘤的病理显示腺癌（HE染色，×200） The pathology of the tumor shows adenocarcinoma (HE staining, ×200)

**2:**
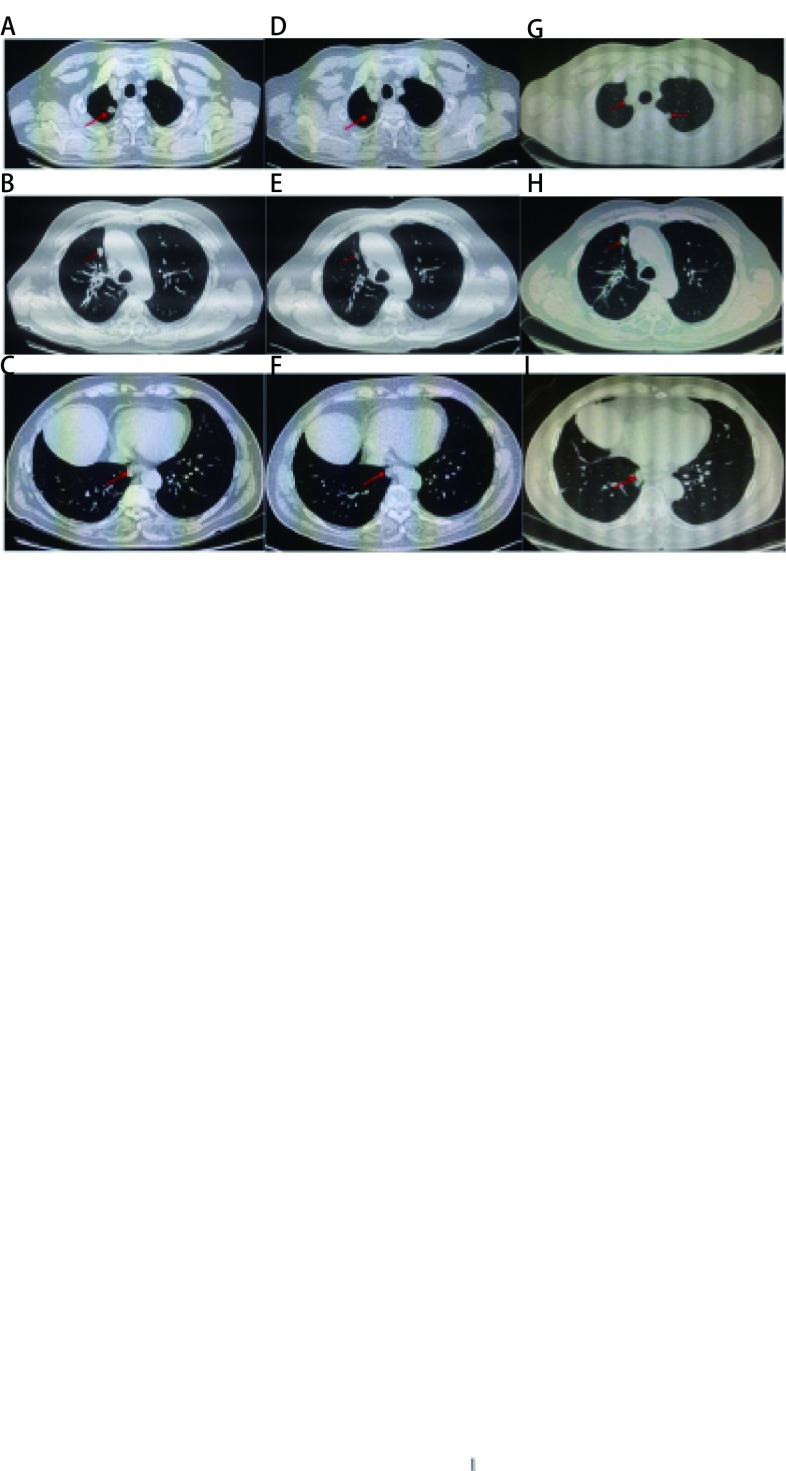
口服安罗替尼前后的胸CT表现。安罗替尼治疗前，2015年11月CT扫描多层面显示双肺多发转移（A-C）。安罗替尼2周期治疗后，2015年12月CT扫描显示转移结节体积变小，并伴有肺空洞形成（D-F）。2017年8月CT扫描显示疾病进展，转移结节数量增多、体积增大（G-I）。 Chest CT scans before and after taking anlotinib.November 2015 CT scans at different layers before anlotinib therapy revealed multiple metastases in bilateral lungs (A-C). After 2 cycles anlotinib treatment, December 2015 CT scans showed that the metastatic nodules became smaller and lung cavity formation (D-F). August 2017 CT images shows disease progression presenting as increased lesion numbers and sizes (G-I). CT: computed tomography.

## 2 讨论

近年来，晚期非小细胞肺癌（non-small cell lung cancer, NSCLC）患者逐渐增多，尽管医学技术发展迅速，治疗方法也明显增多，尤其是靶向药物的应用，然而，目前对于靶向治疗（EGFR/ALK）耐药或化疗失败的三线及以上患者，国内外并没有标准的治疗方案，临床治疗效果也不尽如人意^[1]^。针对这种情况，国内外开展了多项临床研究，希望能够建立晚期NSCLC三线治疗的标准方案，从已经公布的数据来看，ALTER 0303研究是非常令人期待的，ALTER 0303是一项关于安罗替尼作为晚期NSCLC三线治疗的多中心、随机、双盲、安慰剂对照Ⅲ期临床研究，安罗替尼是我国自主研发的新型小分子多靶点酪氨酸激酶抑制剂，与其他TKI不同，可强效抑制VEGFR（VEGFR-1、VEGFR-2和VEGFR-3）、PDGFR（PDGFR-α和PDGFR-β）、FGFR（FGFR-1、FGFR-2、FGFR-3和FGFR-4）和c-Kit等多个靶点^[2]^，具有抗肿瘤血管生成和抑制肿瘤生长的作用，在作用机制上有着明显的优势，因为作用于上述靶点的半抑制浓度（IC_50_）值较低，其安全性更好，安罗替尼的给药方式为12 mg，1次/日，用两周停一周，这种给药方式耐受性更好，并且安罗替尼是口服药物，使用方便，可提高患者生活质量，同时无显著毒性^[3]^。安罗替尼用于晚期NSCLC三线治疗的Ⅲ期研究在2017年美国临床肿瘤学会（ASCO）年会上发布，结果显示安罗替尼组的OS显著长于对照组（9.6个月*vs* 6.3个月，*P*=0.001, 8），并且在PFS（5.4个月*vs* 1.4个月，*P* < 0.000, 1）、客观缓解率（9.2% *vs* 0.7%, *P* < 0.000, 1）和疾病控制率（81.0% *vs* 37.1%, *P* < 0.000, 1）等次要终点上也均显著优于对照组，并且通过扩大的样本量进一步验证安罗替尼的安全性，并未发生治疗相关性死亡事件，主要不良事件包括乏力、高血压、皮肤毒性反应等，通过对症治疗或调低药物治疗剂量等方式，能够得到有效控制，不良反应和预期一致^[4]^。韩宝惠教授在2017年世界肺癌大会（WCLC）上展示了详细的亚组分析结果，入组标准中包括*EGFR*突变耐药和EGFR野生型患者，*EGFR*突变患者要求一代酪氨酸激酶抑制剂治疗进展且接受一线化疗，*EGFR*突变阳性亚组的OS、PFS均显著长于安慰剂组（10.7个月*vs* 6.27个月，*P*=0.022, 7）、（5.57个月*vs* 0.83个月，*P* < 0.000, 1）；野生型亚组的OS、PFS也均优于安慰剂组（8.87个月*vs* 6.47个月，*P*=0.028, 2）、（5.37个月*vs* 1.57个月，*P* < 0.0001）；这说明两组亚型使用安罗替尼都能获得疾病控制和生存获益^[5]^；但是对于安罗替尼的优势人群选择、全程治疗策略的制定等问题，仍有待进一步研究。目前，安罗替尼已进入国家食品药品监督管理总局（CFDA）快速评审通道，有望成为中国NSCLC三线治疗标准。三线治疗是延长NSCLC患者生存的重要手段和有效方法。

本例为EGFR野生型、ALK重排阴性、*KRAS*突变的晚期肺腺癌患者，多线化疗、放疗进展后口服安罗替尼，疗效佳，PFS长达21个月余，不良反应发生程度均较轻微且可以控制，基本不需要进行特殊的药物治疗处理，提高了病人的生活质量。相信未来安罗替尼的上市不仅能够造福于患者，也给临床医生提供了一个新的治疗选择。
